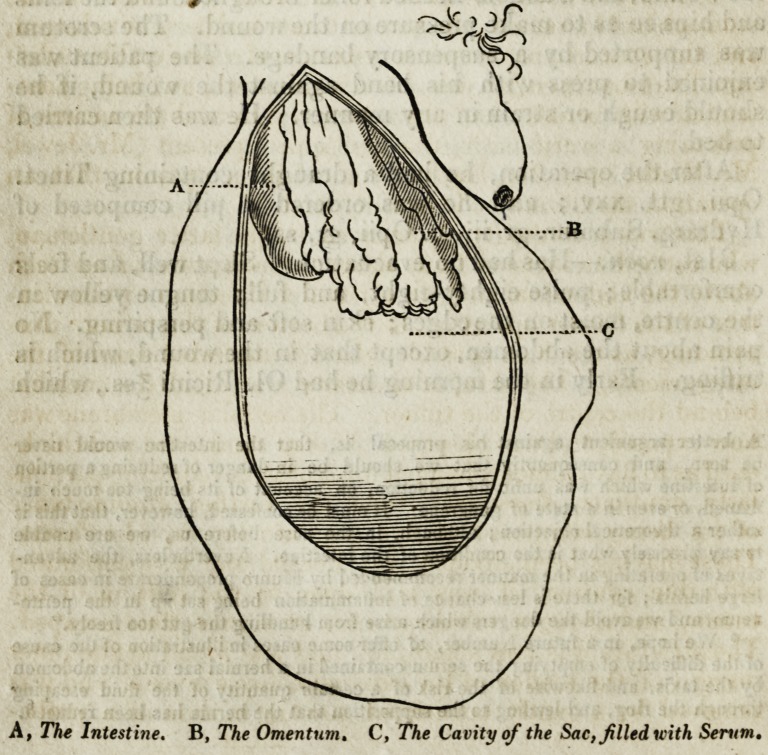# Case of Hernia, in Which the Tumor Was of Very Large Size, in Consequence of an Effusion of Serum into the Sac

**Published:** 1826-07

**Authors:** 

**Affiliations:** the Middlesex Hospital.


*Case of Hernia, in which the Tumor was of very large size, in consequence of an Effusion of Serum into the Sac.* Treated by Mr. Shaw, at the Middlesex Hospital.

May 29, 1826.—James Butler, ætat. forty-three, a bricklayer, was brought into the hospital about twelve o'clock at night, having a large scrotal hernia of the right side. It had come down between three and four o'clock in the afternoon. Several ineffectual attempts to reduce it had been made before he was brought into the hospital.

He states that, ten years ago, while he was ascending a ladder with a large mass of lead on his shoulder, his foot slipped off one of the steps, and in the attempt to recover himself, the rupture came down for the first time. The swelling was then of the size of a pigeon's egg, but in the course of two years it increased much in bulk, notwithstanding that he wore a truss. The hernia has frequently come down since that time, but he could always reduce it easily himself. Yesterday, while carrying a load, a drunken man reeled against him, so as nearly to upset him; and he was conscious of his rupture descending while he was making an effort to regain his balance. He states that he had a copious stool before he was brought to the hospital. He had also been sick, and had vomited. On the abdomen being examined, he complained of great tenderness near the neck of the tumor, and of pain which darted through to his back.

Attempts were made by the house-surgeon, for nearly an hour, to reduce the hernia by the taxis; and thirty ounces of blood were taken from the arm. The efforts at reduction not succeeding, and the symptoms continuing, Mr. Shaw was sent for, at three in the morning. He at first attempted to reduce the rupture in the usual manner; and, from the volume of the tumor, he was of opinion that the application of ice might facilitate this object. No ice, however, could be procured at that early hour, and a solution of nitrate of potass and muriate of ammonia was substituted for it, but without any advantage. A large stimulating injection, containing infusion of senna and salts, was now given. Some sickness and faintness having followed this, Mr. Shaw again tried to reduce the hernia, but without success. Having spent two hours in the attempt, and the symptoms not being yet sufficiently urgent to call for an immediate operation, he left the patient, with directions that he should be put into the warm bath, and bled while there to syncope; and that then the reduction should be again attempted.

May 30th, *eleven o'clock forenoon.*—The tumor is of immense size, extending more than half-way down the thigh. At the lower part of the tumor, the right testicle can be seen projecting; the left testicle forms another projection on the middle of the tumor. The skin is dragged off the penis, the point of which forms a small projection on the upper and lateral part of the hernia. The skin is loose over the tumor, yet the sac can be felt tense and slightly elastic beneath it. The tumor does not lie deeply betwixt the thighs, but the swelling is elevated above the level of the belly. This is caused by the tucking of the external abdominal ring upon the neck of the hernia, making the lower part of the tumor tilt upwards. The tumor ends abruptly just opposite to the ring, there being no obliquity of the swelling in the direction of the anterior spinous process of the ilium. The patient points to this part with his finger as the seat of pain, and "what binds him."

It is remarkable that the size of the tumor has increased considerably since three this morning. This circumstance was even observed by the patient himself. Mr. Shaw afterwards stated, that, when he first saw the patient with such an enormous tumor, he suspected that it was an old irreducible hernia, containing a considerable quantity of omentum or intestine, and that a new portion had been pushed down, and become strangulated. But, when the patient, who is a very intelligent man, told him that, previously to yesterday, nothing was in the scrotum except the testicles, he conjectured that the tumefaction might be formed by the gut being suddenly filled with flatus, and that, under such circumstances, its size might be reduced by the application of cold. But it was distinctly proved, during the operation, that the great bulk of the tumor was formed, not by the contents of the gut, but by the secretion from its peritoneal surface, and from that of the sac.

The surgeons, having again employed the taxis without diminishing the bulk of the hernia, and the symptoms continuing, though not very urgent, they considered it most prudent to make no further use of the taxis, but to have recourse to the operation. It was thought probable that, by relieving the stricture produced by the external abdominal ring, they might be able to reduce the hernia without opening the sac. As the hernia was unusually large, it was determined to make only a small incision, so as to expose the ring, and not to divide the neck of the sac, unless it should be found necessary.

The operation was commenced by Mr. Shaw carrying his scalpel quickly through a portion of integument which was pinched up directly over the neck of the tumor: in this way he made at once an incision of three inches long in the line of the spermatic cord, the centre being exactly over the stricture. The layers of cellular membrane were divided with rapidity, so long as they were loose and could easily be seized with the forceps; but, when the neck of the tumor was exposed, the layers of fascia became more firmly adhering, and they were more cautiously raised, the director was insinuated beneath them, and they were divided by running the bistoury along the groove. In this manner the tendon of the external abdominal muscle was exposed. Several firm bands of fascia, which were formed like cords over the external ring, were divided with the bistoury. A smooth uniform fascia was now brought into view, apparently continuous with the columns of the ring. This being opened, the director was pushed under the indented margin of the ring. There was considerable difficulty and great caution required in effecting this, on account of the bulging of the tumor, and the depth and tightness of the ring. When the division of the ring was made by the bistoury, the inferior edge of the internal oblique muscle could be perceived, and a fascia in which we could discover the fibres of the cremaster muscle. There was obviously great relaxation produced in the neck of the hernia, by the division of the external abdominal ring, and it was therefore attempted to reduce the tumor. But this trial was not persevered in, because it appeared that the neck of the sac also produced a certain degree of stricture. Accordingly, the fibres of the cremaster muscle, and its fascia, were cleared away, and the peritoneal sac was exposed. It was pinched up with the forceps, and opened by cutting with the knife carried horizontally. The director was now introduced as far as the neck of the sac, and then the bistoury, being run along the groove, was raised, so as to divide the neck of the sac. The extent of the incision of the peritoneal sac did not exceed an inch and a half.* The finger of the operator

* It was hoped that the intestine might have been reduced without opening the sac: when, however, it was found necessary to open it, the aperture was made as small as possible. Perhaps the following remarks, made by Mr. Bell in his clinical lecture on a case resembling this, will show the grounds on which the operator was unwilling to open the sac. "The operation performed in this case was originally recommended by Dr. Alexander Monro, who was of opinion that the danger of the operation for hernia resulted from the incision of the peritoneum, and the admission of air into its cavity. The Doctor carried this idea so far as to propose to perform the operation of Cæsarean section under water: indeed, so wedded was he to this view of the matter, that he relates the case of a wound of the pericardium with a red-hot poker, as an instance of what he conceived to be the effect of air admitted into a cavity. But, although his reason for operating in this way be untenable, the proposal is not to be rejected on that account.

could now be admitted, and it was ascertained that the stricture was removed.

Mr. Shaw now commenced the reduction of the hernia, by compressing the tumor, in order to evacuate its contents. During this attempt, a portion of omentum (healthy in appearance) was pushed out of the sac; and the assisting surgeon placed his finger upon this, to prevent any more from escaping. Meanwhile the tumor was compressed, yet it was not much diminished in its volume. On ceasing to close with the fingers the aperture through which the omentum was protruded, and again compressing the tumor, a jet of serum was forced from the wound, and continued to flow to the extent of more than a pint. When the effusion was evacuated, the size of the tumor was found to be very much diminished, there being left only a small turn of intestine, which was not very full, and covered on its fore part by the portion of omentum which protruded. The intestine was reduced with great ease, and the omentum was pushed up afterwards.*

The lips of the wound were brought together by two ligatures and adhesive plasters. Compresses were then laid over the wound, and a double-headed roller brought round the loins and hips so as to make pressure on the wound. The scrotum was supported by a suspensory bandage. The patient was enjoined to press with his hand against the wound, if he should cough or strain in any manner. He was then carried to bed.

After the operation, he had a draught containing Tinct. Opii, gtt. xxv.; and he was ordered a pill composed of Hydrarg. Submur. gr. iij. c. Opii, gr. ss.

31st, *noon*.—Has had no evacuation. Slept well, and feels comfortable; pulse eighty-eight, and full; tongue yellow in the centre, moist on the edges; skin soft and perspiring. No pain about the abdomen, except that in the wound, which is trifling. Early in the morning he had Ol. Ricini ℥ss., which

A better argument against his proposal is, that the intestine would never be seen, and consequently that we should be in danger of reducing a portion of intestine which was unfit for reduction, on account of its being too much inflamed, or even in a state of gangrene. It must be confessed, however, that this is rather a theoretical objection; although, in the case before us, we are unable to say precisely what is the condition of the intestine. Nevertheless, the advantages of operating in the manner recommended by Munro preponderate in cases of large hernia; for there is less chance of inflammation being set up in the peritoneum, and we avoid the dangers which arise from handling the gut too freely."

* We hope, in a future Number, to offer some cases in illustration of the cause of the difficulty of emptying the serum contained in a hernial sac into the abdomen by the taxis, and likewise of the risk of a certain quantity of the fluid escaping through the ring, and leading to the supposition that the hernia has been reduced.

has had no effect. He is ordered to have another dose of castor-oil in the course of an hour.

June 1st.—Yesterday, about two o'clock, he had a plentiful stool, and he has had several evacuations since. Pulse eighty-six, and full; tongue moist; skin perspiring. He slept comfortably, and feels easy.

June 2d.—To-day the wound was dressed: the edges are in close apposition, and promise to unite by the first intention. All the symptoms continue favourable. His bowels have been well opened since the last report.

June 6th.—We have not thought it necessary to give a report each day of this man's condition, because he has continued quite free from unfavourable symptoms since our last report. To-day (being the eighth after the operation,) the wound was found so completely adhering, that only a line marked the place of the incision. The adhesive straps were thought no longer necessary; the compress and roller are to be continued.

10th.—Free from all complaint.

The subjoined cut will give a general idea of the *relative* size and situation of the parts.

## Figures and Tables

**Figure f1:**